# The Role of the Autonomic Nervous System on Cardiac Rhythm during the Evolution of Diabetes Mellitus Using Heart Rate Variability as a Biomarker

**DOI:** 10.1155/2019/5157024

**Published:** 2019-05-09

**Authors:** Alondra Albarado-Ibañez, Rosa Elena Arroyo-Carmona, Rommel Sánchez-Hernández, Geovanni Ramos-Ortiz, Alejandro Frank, David García-Gudiño, Julián Torres-Jácome

**Affiliations:** ^1^Universidad Nacional Autónoma de México, Centro de las Ciencias de la Complejidad, Circuito Mario de la Cueva 20, Insurgentes Sur, Delegación Coyoacán, C.P. 04510 Cd. de México, Mexico; ^2^Benemérita Universidad Autónoma de Puebla, Instituto de Fisiología, 14 Sur 6301, Colonia Jardines de San Manuel, C.P. 72570 Puebla, Pue., Mexico; ^3^Benemérita Universidad Autónoma de Puebla, Facultad de Ciencias Químicas, 18 sur y avenida San Claudio colonia Jardines de San Manuel, C.P. 72570 Puebla, Pue., Mexico; ^4^Universidad de Puebla, Escuela de Ciencias Químicas, Colonia Guadalupe Hidalgo, Puebla, Pue., Mexico

## Abstract

Heart rate variability (HRV) is highly influenced by the Autonomic Nervous System (ANS). Several illnesses have been associated with changes in the ANS, thus altering the pattern of HRV. However, the variability of the heart rhythm is originated within the Sinus Atrial Node (SAN) which has its own variability. Still, although both oscillators produce HRV, the influence of the SAN on HRV has not yet been exhaustively studied. On the other hand, the complications of diabetes mellitus (DM), for instance, nephropathy, retinopathy, and neuropathy, increase cardiovascular morbidity and mortality. Traditionally, these complications are diagnosed only when the patient is already suffering from the negative symptoms these complications implicate. Consequently, it is of paramount importance to develop new techniques for early diagnosis prior to any deterioration on healthy patients. HRV has been proved to be a valuable, noninvasive clinical evidence for evaluating diseases and even for describing aging and behavior. In this study, several ECGs were recorded and their RR and PP intervals were analyzed to detect the interpotential interval (ii) of the SAN. Additionally, HRV reduction was quantified to identify alterations in the nervous system within the nodal tissue via measuring the SD1/SD2 ratio in a Poincaré plot. With 15 years of DM development, the data showed an age-dependent increase in HRV due to the axon retraction of ANS neurons from its effectors. In addition, these alterations modify the heart rhythm-producing fatal arrhythmias. Therefore, it is possible to avoid the consequences of DM identifying alterations in SAN previous to its symptomatic appearance. This could be used as an early diagnosis indicator.

## 1. Introduction

Heart rate variability (HRV) results from the interaction between the ANS and the SAN activity [1]. Measurements of the fluctuations within HRV are a noninvasive method used to evaluate the nervous system under physiological and pathological conditions [2]. Such fluctuations arise from the regulation between the sympathetic and parasympathetic nervous systems, branches of the ANS [3] which have been evaluated with spectral analysis and time series methods [4]. The time series analysis of HRV is considered to be a trustworthy biomarker to evaluate diseases and even for describing aging and behavior [5]. For DM, HRV is an early biomarker for determining the progression of the illness [6]. Arroyo-Carmona et al. [2] used the RR time series of several electrocardiograms (ECG) for defining the variability in HRV. An ECG is the record of the electrical activity of the heart tissue, each of which is represented by different waves with distinctive amplitudes and durations. The ECG morphology is the result of the ANS and SAN activities and can be classified into two groups: the positive deflections and the negative deflections. The positive deflections encompass the *P*, *R*, and *T* waves. The *P* wave represents the electrical activity of both atrial nodes, the *R* wave represents the ventricular depolarization, and the *T* wave represents ventricular repolarization [6]. The negative deflections include the *Q* and *S* waves. The most commonly taken into account intervals for measuring HRV are the *R*-*R*, *Q*-*T*, and *P*-*R* intervals and the QRS complex [7]. The pacemaker of the heart generates the electrical activity responsible for the intrinsic heart rate which is in the SAN. Although its depolarization cannot be seen on the ECG, the shape of the *P* wave could give an idea of its electrical behavior [8]. HRV has been thought to be solely the variability of the ANS and has been therefore statistically analyzed for serving only as a predictor of the regulation of ANS. However, as recent studies reveal that the SAN also has its own variability, it is of paramount importance to separately evaluate the correlation of both oscillators in order to use HRV to be an even better biomarker for evaluating diseases and even for describing physiological conditions such as aging and behavior. The aim of this study is to prove HRV as a clinical biomarker for framing the changes during the progression of DM. For this purpose, an animal model of chronic diabetes type 1 in mice (cDM) was used.

## 2. Material and Methods

### 2.1. Animal Model (Diabetes Mellitus Type 1)

Adult male mice CD1 8 weeks old with 33 g of weight on average were used in this study. All the animals were maintained with a 12 : 12 h light-dark cycle (7:00-19:00) and allowed free access to LabDiet 5001 pellets and water. The cDM model was induced with streptozotocin at 120 mg/kg weight, and it was used thereafter at 10 and 20 weeks of induction DM (cDM model) [2]. All methods used in this study were approved by the Animal Care Committee of Instituto de Fisiología Celular, Universidad Nacional Autónoma de México. Animal care was in accordance with the “International Guiding Principles for Biomedical Research Involving Animals,” Council for International Organizations of Medical Sciences, 2013 [2].

### 2.2. Diabetes Mellitus Evaluation: Electrocardiogram

The electrical activity was recorded at 8 weeks of age just before the DM induction; ten and twenty weeks following induction of DM, the parameters were compared with control. The mice were anesthetized with pentobarbital sodium 0.63 g/kg i.p. and placed in supine position for 30 minutes of ECG recordings. The bipolar ECGs were recorded with subcutaneous needle electrodes in configuration lead I. The electrodes were placed right and left in the fourth intercostal space. The ECG signal was amplified 700 times and filtered at 60 Hz. The signal was recorded on a PC at a sampling frequency of 1 KHz and analyzed offline with Clampfit® program (Molecular Devices). For the HRV analysis, the 30-minute long ECG recordings were cut into 5-minute series [7]. Subsequently, a hundred RR, PP, and action potential intervals were randomly selected. The intervals were measured between consecutive beats. All mice were continuously monitored to guarantee adequate ventilation and temperature.

#### 2.2.1. Intrinsic Heart Rate Variability Recording of the Pacemaker

The nodal tissue was prepared as previously reported by Arroyo-Carmona et al. [2], and spontaneous electrical activity was recorded using the conventional microelectrode technique. The interpotential interval (ii) was measured for all zones of the pacemaker [3].

#### 2.2.2. Heart Rate Variability Evaluation

For the evaluation of HRV, two approaches were used. The first was used to fit the tendency of the power spectral density (PSD), for determining the behavior of the time dependence within HRV. The second item was used for determining the magnitude of variability which was calculated SD1, SD2, and intrinsic heart rate variability using the Poincaré plot. For the construction of the Poincaré plot, the RR and PP intervals were used, which are the time between the maximum of the corresponding waves on the ECG and the interpotential interval of the pacemaker.

The Poincaré plot represents the RR_*i*+1_ interval as a function of the previous RR_*i*_ interval. The heart rate is the inverse RR interval. SD1 is the standard deviation of the distances between all points of the Poincaré diagram and the RR_*i*+1_ = RR_*i*_ line. SD2 is the standard deviation of the distance between all points of the Poincaré diagram and the ${\text{RR}}_{i+1}=-{\text{RR}}_{i}+2{\bar{\text{RR}}}_{i}$ line where ${\bar{\text{RR}}}_{i}$ is the average value of all RR_*i*_ [2]. iHRV is the SD1/SD2 ratio which is the value that suggests the delicate equilibrium between the sympathetic and parasympathetic systems of the heart [8]. Also, the Poincaré diagram was made with PP_*i*_ intervals and interpotentials (ii); the first reflected the auricular electrical activity. For the evaluation, the behavior of the whole correlation function used the power spectrum temporary time series RR and PP intervals of ECG of several stage ages of mice.

### 2.3. Data Analysis and Statistics

#### 2.3.1. Poincaré Plot

All the data are presented as mean ± standard error. The *t*-test was used for data analysis; the values were considered statistically significant if the value was lower than 0.05 which is denoted with ∗. The analysis was made in the OriginPro version 8.0 from Lab Corporation.

The distances for the obtained SD1 and SD2 were calculated with

(1) $$\begin{array}{c} \sqrt{{\left(\frac{{\text{RR}}_{i}-{\text{RR}}_{i+1}}{2}\right)}^{2}}, \end{array}$$

(2) $$\begin{array}{c} \sqrt{2{\left(\frac{2{\bar{\text{RR}}}_{i}-{\text{RR}}_{i}-{\text{RR}}_{i+1}}{2}\right)}^{2}}. \end{array}$$

With all distances in equations (1) and (2), the SD1 and SD2 standard deviations were determined, respectively.

#### 2.3.2. Power Spectral Analysis

In analyzing the frequency content of the signal *f*(*t*), one might like to compute the ordinary Fourier transform *F*(*w*); however, for many signals of interest, the Fourier transform does not formally exist. Because of this complication, one can work as well with a truncated Fourier transform where the signal is integrated only over a finite interval [0, *T*]:

(3) $$\begin{array}{c} F\left(w\right)=\frac{1}{\sqrt{T}}\int_{0}^{T}f\left(t\right)e^{-it\omega }dt. \end{array}$$

This is the amplitude spectral density. Then, the power spectral density (PSD) can be defined as [2, 3]

(4) $$\begin{array}{c} S\left(\omega \right)=\underset{T\to \infty }{\lim}E\left[{\left|F\left(w\right)\right|}^{2}\right]. \end{array}$$

By fitting the tendency of the PSD, it is possible to characterize the behavior of a system; for example, if *f*(*t*) is a white noise signal (which is characterized for having all possible frequencies in the same fraction), the tendency will be a line with zero slope (*m*). Other examples consist in signals known as scale invariant which have a slope depending on the frequency (*w*) as 1/*w* [4].

#### 2.3.3. Immunofluorescent Staining

Indirect immunostaining was analyzed using confocal microscopy (Confocal Olympus FV1000, Olympus America Inc.). SA nodes were isolated as mentioned above, embedded in Tissue-Tek (Sakura), frozen, and cut coronally into 5 *μ*m thick slices beginning from the endocardium. The antibodies used were anti-tyrosine hydroxylase (1 : 250 rabbit polyclonal antibody; Millipore Corporation) and CY5 (1 : 200, rabbit polyclonal antibody; Jackson ImmunoResearch Laboratory Inc.).

## 3. Results

### 3.1. Development of the cDM Model Compared with Human

For relating ages between the animal model and human, a scale was constructed according to Dutta [9] and Koening [6]. Mouse adulthood (*n* = 15), as related to human age, is eight weeks compared with humans, which is at 17 to 22 years of age, according to Dutta [9]. Mice at eighteen weeks are 30 to 35 years old (*n* = 13) [9]; they must have 8 years of development with DM ten weeks after induction DM (*n* = 13). The mice at twenty-eight weeks are 40 to 45 years old (*n* = 10), and the DM model has chronic diabetes with 15 years development of DM twenty weeks after induction (*n* = 13).

### 3.2. Heart Rate

The heart rate was described using common RR intervals; in age three in control mice, the mean for adulthood (17-22 years human age) was 284 ± 46 bpm; the mean data showed an increase by 31%; at eighteen weeks or 30 years old and at twenty eight weeks old or 40 years old, the mice increased by 34% (Table 1). The heart rate decreased by 16% in early DM (ten weeks of development) compared with control and increased by 10%, beside adulthood. On the other side, the animals with chronic DM (twenty weeks of development) had an increase by 16% compared with control and 43% with adulthood (Table 1).

Also, the heart rate was characterized with PP intervals. In the same way, the heart rate increased with age; in adulthood, it was 279.06 bpm, 329.6 bpm at 30 years of age, and 379 bpm at 40 years of age. In the early eight years of development of diabetes, the heart rate decreased by 10% compared with control and did not change with adulthood. Subsequent of fifteen years of developing diabetes, the mice did not present changes compared with control animals, but compared with that at adulthood, the heart rate has an increase by 35%.

As expected, the pacemaker presented a low rate of firing by aging, and the frequency intrinsic at 30 years was 218 (ii/min) and 190 (ii/min) at 40 years old, inasmuch as the autonomic nervous system was unmodulated. The animals with diabetes in the early and chronic stages augmented rate firing at 258 and 208 respectively, although following the rule of decrease in firing by aging (see Table 2).

### 3.3. Heart Rate Variability

#### 3.3.1. Heart Rate Variability

For analysis of HRV, we have studied two different types of time series, the PP and RR series obtained from the ECG. Each of them is from three control cases with adulthood, 30 and 40 years old, and two from a group of ill subjects with the same ages as in the control groups.

**Figure 1:**
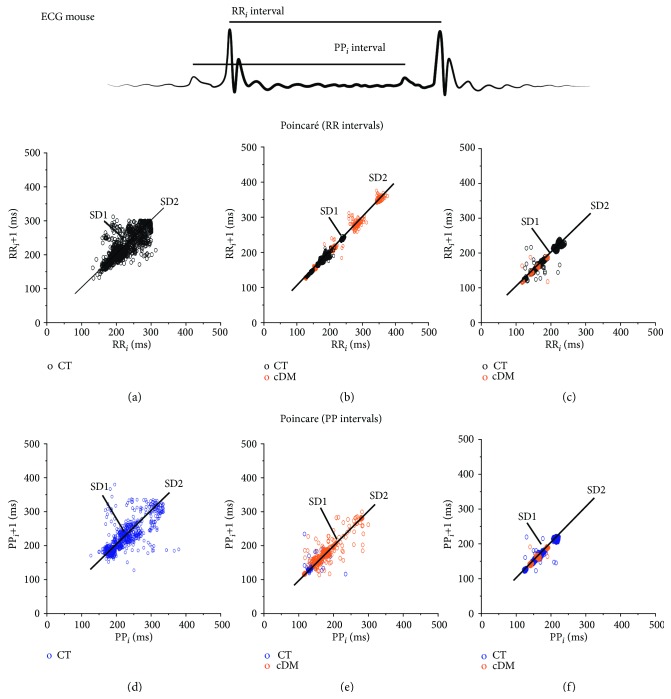
Age and development of diabetes mellitus alter the heart rate variability in the cDM model. (a, d) In adulthood, the morphology of the Poincaré plot is an ellipse with axe major SD2 and axe minor SD1, and the SD1/SD2 index was 0.5 for the PP interval and for RR interval SD1/SD2 = 0.43. (b, e) At 30 years of age with 8 years' development of DM, the data showed the function of the parasympathetic system. (c, f) At 40 years of age with 15 years' development of DM, the data showed that the nervous system ceased to function, and there were no changes in SD1 and SD2. Index with respect to control.

**Figure 2:**
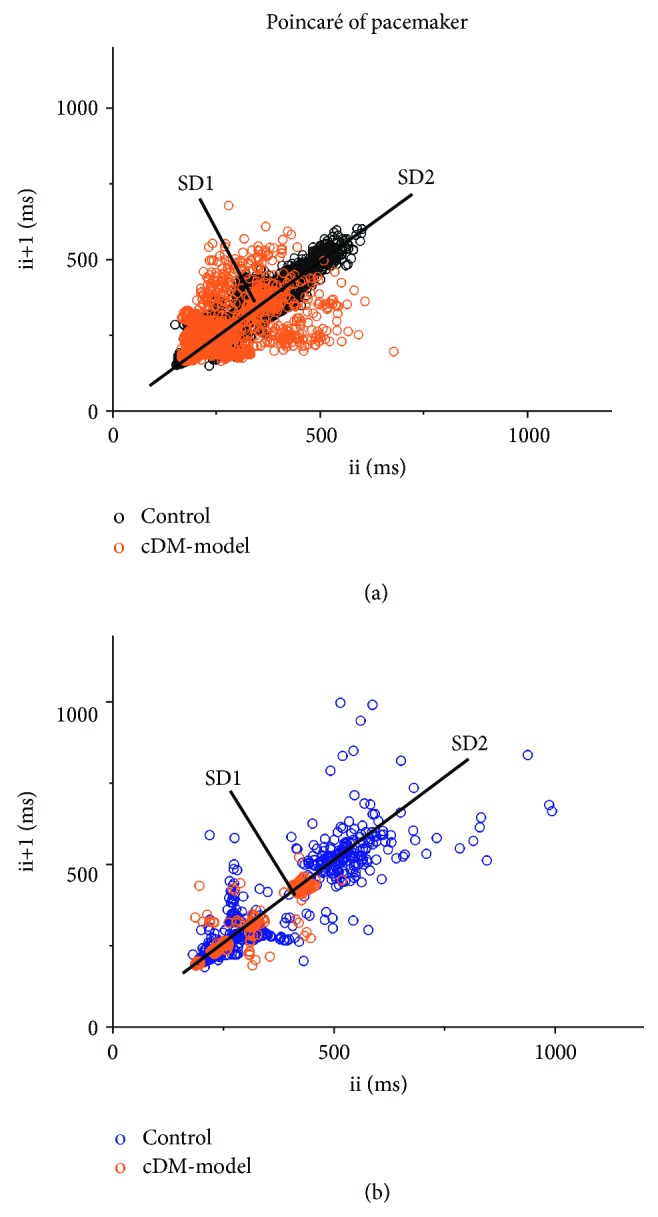
Pacemaker without the influence from the nervous system increases variability. The interpotential (ii) of the pacemaker in the control (a) at 30 years of age and (b) at 40 years of age. The development of diabetes mellitus increases both variability and frequency (a) at 8 years' development and (b) at 15 years' development of DM.

*(1) The Poincaré Plot*. In the Poincaré graph of RR intervals, during adulthood, variability of SD1 = 12, SD2 = 28, and ratio of 0.43 similar to humans was observed [10, 11]; the variability decreased by age, at eighteen weeks of age variability decreased to SD1 = 2, and at twenty-eight weeks of age variability was SD1 = 1, while SD2 only changed in the last age, SD2 = 1.3 (Table 3 and Figure 1). When using PP intervals for the Poincaré plot, the HRV decreased by age in both SD1 and SD2; the literature suggests for humans [1]. The HRV in adulthood was observed with SD1 = 19 and SD2 = 35 and ratio of 0.54; when the animals are 30 years old, they show a decrease of 60% and 32%, whereas the mice with 40 years of age have 1.1 and 1.1 for SD1 and SD2, respectively (Table 3 and Figure 1). Diabetes in the early stages altered the delicate equilibrium of the autonomic nervous system, while SD1 increased with 8 and 10 and SD2 with 46 and 56 in both system RR and PP intervals, respectively. When comparing the variability of the animal model with diabetes and adulthood, SD1 is low and SD2 rises (see Table 2 and Figure 2). After fifteen years of development of diabetes in mice, the variability decreases in RR intervals SD1 = 0.6, SD2 = 0.6, and PP intervals SD1 = 1.2, SD2 = 0.9 in addition, SD1 and SD2 are similar, namely, nondynamic systems (Table 3 and Figure 2).

**Figure 3:**
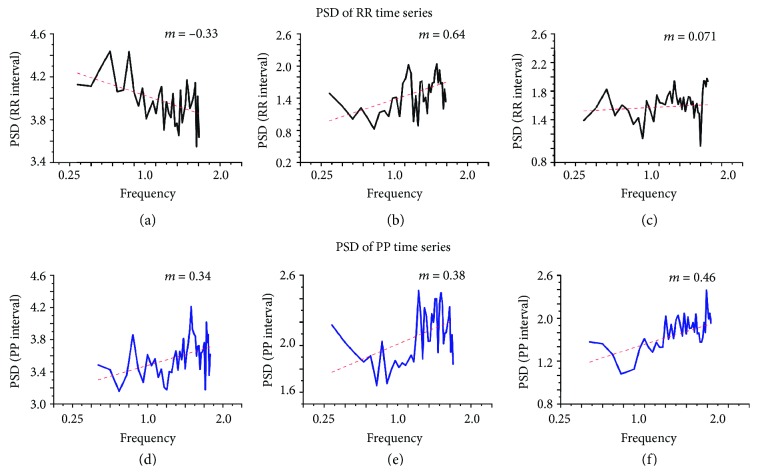
Power spectral analysis by age. Control subjects (*n* = 15) with increasing age from RR and PP time series. The slope varies with age with an erratic behavior in RR intervals (a, b, c). The slope of the fit line increases according to age in PP intervals (d, e, f).

**Figure 4:**
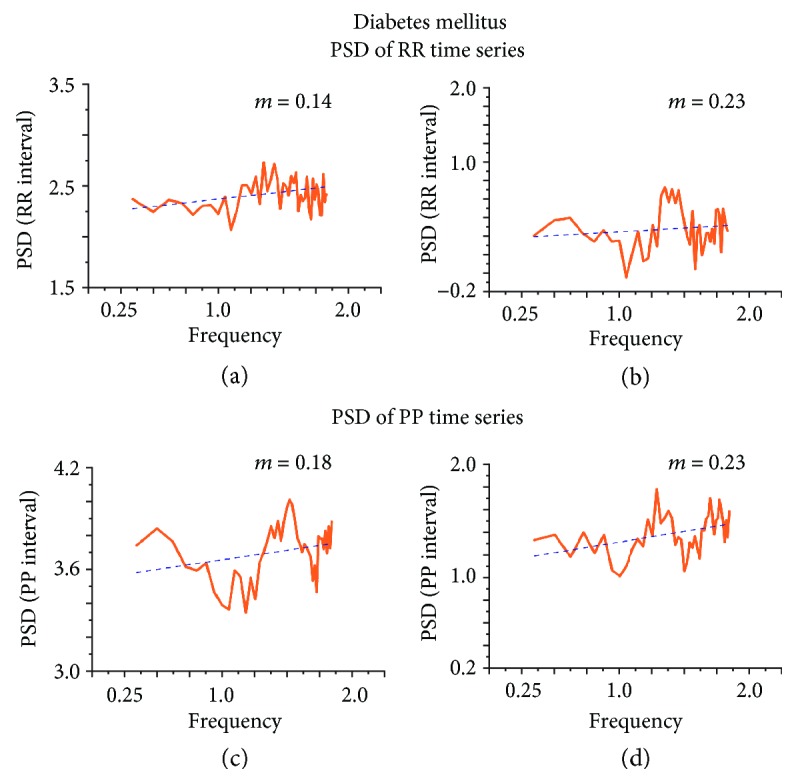
Power spectral analysis of the development of diabetes. The diabetes from RR time series (a, b) and PP time series (c, d) was a slope of approximately zero (*n* = 13) indicating loss of natural frequency PSD.

*(2) The Power Spectral Density Analysis*. In the PP time series, according to age change, it was observed that the tendency of the PSD increases [12, 13], which indicates alterations in the rigidity of the system, since it is favoring a specific frequency (Figures 3(d)–3(f)), which refers to the diabetes cases (Figure 4); although the slope is not zero, it is clearly closer to this value than in the control cases. This would mean that the heart is losing its characteristic frequencies and is approaching white noise (which has all frequencies indistinctly).

For the time series of RR of the control mice, in terms of age, nothing can be said with certainty, because the adjustment of the PSD has no order in the slopes (*m*); in fact, one of them is practically zero, which is what we would expect in case of diabetes [14] (see Figures 3 and 4). In cases of diabetes, there is a slope very close to zero, which reinforces the previous results (see Figure 4).

### 3.4. Heart Rate Variability of the Pacemaker

The HRV intrinsic of the pacemaker using interpotential intervals (ii) showed greater variability than all intervals, in adulthood SD1 = 58% and SD2 = 25% major than RR intervals; however, HRV decreased by age at 30 years old, SD1 = 67% and SD2 = 50%, and at age 40 years old SD1 = 1700% and SD2 = 3200% were decreased. The cDM model with 8 years development showed a decrease in SD1 = 48%, increase in SD2 = 60%, and with 15 years development a decrease in SD1 = 1600% and SD2 = 3800% (see Figures 1 and 3(e)–3(f) and Table 2).

**Figure 5:**
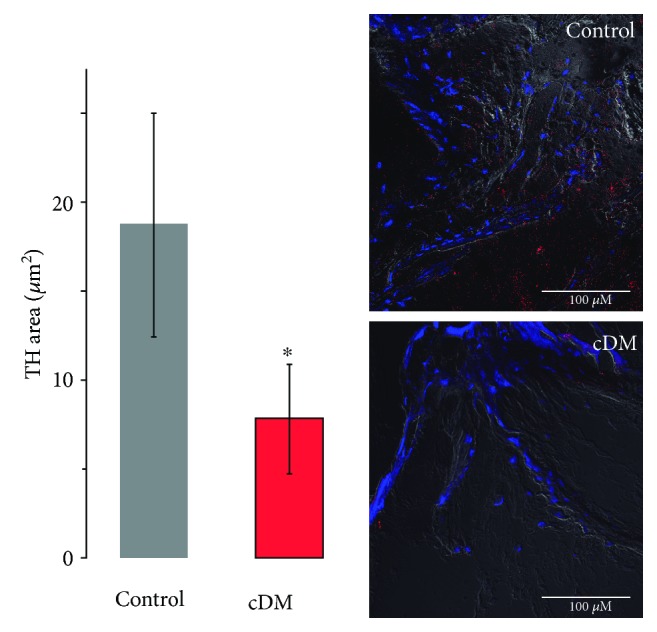
Decrease in the nervous system with 15 years' development of cDM. Average of nodal tissue staining with antibody of tyrosine hydroxylase (red) in the control and decreased signal in the nodal tissue of cDM mice (*n* = 3, Student *t*-test: *p* < 0.05^∗^ vs. control).

In the same way, the Poincaré plot of the pacemaker showed an increase in the parasympathetic system SD1 = 61 and a concomitant lowering decrease in the sympathetic system SD2 = 45 at the 8-year development of DM compared with control SD1 = 10 and SD2 = 61, while the 15-year development of DM had an increase, SD1 = 13, SD2 = 14, compared with control SD1 and SD2 15. The index SD1/SD2 at 15 years of development was 1 and cDM 0.9 (Figure 2 and Table 2); this result was consistent with the decrease in tyrosine hydroxylase staining in the nodal tissue (Figure 5).

## 4. Discussion

DM is a higher factor of risk associated with cardiovascular mortality, in accordance with glucose management (diabetes mellitus type 1 or diabetes mellitus type 2) and other factors such as dyslipidemia, hypertension, microvascular complication, and duration DM [7]. However, the diagnosis for the DM type 2 is not timely; consequently, the poor glycemic control and combination with other factors could be manifest as tachycardia and development of “silent” myocardial infarction [15]. Furthermore, the telemonitoring of electrical activity of the pacemaker in patients at a very high risk developing fatal arrhythmias has helped to diminish atrial fibrillation (AF) and ventricular tachycardia episodes significantly [16]; also in patients with pacemaker, it is a powerful diagnostic tool for predicting heart failure and reducing its hospitalization [17]. Thus, a method that is not invasive for diagnosis and prognosis is necessary, for lessening side effects as cardiovascular disease mortality. In this article, we proposed to use the interaction between ANS and SAN as a tool to inform above physiological and pathological conditions of body health.

The heart has electrical activity intrinsic with a physiological variability as an oscillator (Figure 2); SD1 represents the variability for a short time between the *i*-interval and the *i*-interval + 1 while SD2 is the variability of change with respect to average variability. The data in Tables 2 and 3 showed that pacemaker variability has an extensive range of frequencies to characterize its stable state; this means that the pacemaker variability could be modified by any perturbation outside its frequency range [3]. A physiological perturbation on the pacemaker is the ANS. In this case, for RR and PP intervals of ECG, it would be the interaction of the SAN-parasympathetic system as SD1, SAN-sympathetic as SD2, and SD1/SD2 as the relation between two oscillators (Figure 1). In the same way, the interaction between ANS and heart intrinsic activity is altered during aging similar to diabetes [18]; this involves fragility in the interaction between both SD1/SD2 (Tables 2 and 3).

The mice with early diabetes showed alterations in the delicate balance of the autonomic nervous system, such as SD1 decrease and SD2 increase added to resting tachycardia present in the pacemaker, suggesting cardiovascular autonomic neuropathy (CAN) in the early stages [19]. This data could be supporting the information about a poor diagnosis of diabetic autonomic neuropathy in early diabetic patients [20]. These patients may have only the silent AF as subclinical disease [21]. Other signs of a relationship with AT (atrial tachycardia) are changes in *P*-wave duration and dispersion [20]. This information proposed that the heart is the first organ injury for diabetes, the pacemaker primarily. Likewise, as diabetes progresses, the relationship with CAN is more evident. The mice with a fifteen-year development of diabetes showed resting tachycardia both in the heart with ANS and intrinsic pacemaker (Tables 1 and 3, respectively); additionally, these mice showed denervation in the pacemaker tissue (see Figure 5). The highest resting heart rate abnormality is related to damage in the parasympathetic system in the early stage of development of CAN [22].

A strategy to detecting CAN could be through reduction in HRV, measured by power spectral analysis; in healthy humans, beat-to-beat variation is recorded during inspiration and expiration, which is driven by sympathetic and parasympathetic activity to obtain three components of the power spectrum: (a) the thermoregulatory activity is reflected in very low frequency (0.003–0.04 Hz) or sympathetic activity; (b) the baroreceptor activity is reflected in low frequency (LF; 0.04–0.15 Hz) or a mixture of parasympathetic and sympathetic activity; and (c) it reflects respiratory activity expressed in high frequency (HF; 0.15–0.4 Hz) or parasympathetic activity [19, 22]. In this case, in the animal model, any cardiac autonomic cannot performed, and it has different component values in the frequencies compared to humans (see Figures 3 and 4).

In this paper, for measuring the reduction in HRV, the characterization in the behavior of the time dependence of PSD is proposed. The analyses of RR time series showed that the frequencies with major involvement in adulthood were 0.43 and 0.52 Hz; in mice with 30 years of age, the frequency is greater than 1.09 Hz; and at 40 years of age, the slope is near zero. The last point means that the time series is composite by all frequencies similar to a white noise (Figures 3(a)–3(c)). This suggests that the robustness of RR intervals decreases in the process of aging.

However, in the HRV of PP time series, the frequency major than 1.27 Hz was the biggest participation, a slope of PSD was rising by age, and the analysis of PP time series allowed characterizing the frequencies during aging; for example, the slope is not zero (Figures 3(e) and 3(f)) [1]. The intrinsic activity or pp time series do not lose robustness.

In contrast, DM showed that both intervals (RR and PP) were white noise; this implies that the system loses robustness by diabetes (Figure 4). The characterization of HRV by this method is independent of maneuvers that implied to control more than one variable similar to thermoregulation, circadian rhythm, or respiration; in addition, several species could be used.

Also, HRV was quantified with a Poincaré plot analysis where the sympathetic-heart (SD2) interaction is double the parasympathetic and heart (SD1) interaction [23]; consequently, the Poincaré index was ~0.5 in adulthood (see Table 3). As age advances, this delicate relation decreases in SD1 but SD2 had changes after 30 years of age; this suggests that SD2 has a major participation in healthy conditions.

The results presented in alterations of HRV by early diabetic have been associated to the interaction lowering of the parasympathetic system and increase in the sympathetic system on electrical activity of the heart, without apparent shifts in the vascular system and peripheral nervous system (Figure 3). Chronic DM decreases by 20 times in SD1 and 46 times in SD2 with a ratio of 1 (see Figure 1 and Table 3). This could mean that the interaction between the ANS and heart was lost, so heart rate variability depends only the pacemaker (intrinsic activity) [3, 24] or the interaction between the sympathetic and parasympathetic systems is equal (autonomic balance) [25], such as the Poincaré plot of interpotential data which showed an index of 1 at fifteen years of DM (Table 2). According to the data shown in Figure 5, the nervous system of pacemaker tissue decreases in chronic diabetes and consequently increases the risk of CAN, such as the Poincaré plot of interpotential data which showed an index of 1 at fifteen years of DM (Table 3). According to the data shown in Figure 5, in the pacemaker tissue, the nervous system decreases in diabetes increasing the risk of CAN after fifteen years with DM. The variability of PP intervals allowed observing the sympathetic and parasympathetic systems' interaction due to aging and development of DM, such as the changes in SD1, SD2, and SD1/SD2 for aging SD1, SD2, and ratio decrease, while these parameters presented minor robustness in DM; on the other hand, the variability of RR intervals does not observe this correlation, explicitly with RR interval variability which does not sense changes in SD2 by aging (see Table 2).

It is known that dyslipidemias rise in the nervous system in SAN [3]. Thus, our cDM model may also be attractive for researches with new pharmacological treatments like GLP-1 and defibrillator [26]; both treatments could be anticipated of heart failure with dyslipidemia in the first stage of diabetes, decrease hospital admission and death in diabetic patients [16], and reduce microvascular complications [24]. For this reason, the development of an animal model like cDM with pharmacological chronic therapy of GLP-1 and monitoring your HRV would improve macro- and microvascular side effects inclusive of fatal cardiovascular events [27].

## 5. Conclusion

In the early stages of development of DM, the influence of the nervous system allowed maintaining the balance of an elliptical shape in the Poincaré plot; however, in diabetes mellitus by 15 years of development in SAN, this balance is altered in the PP interval Poincaré plot and PSD. However, in SAN, there is an increase in the variability at 8 and 15 years' development of DM. Therefore, it is important to observe the variability in PP intervals and increase changes in the rhythm and cardiac arrhythmias; finally, the proposal to use HRV for diagnostic and prognostic side effects by alterations in the rhythm producing fatal arrhythmias is very useful. It is also important that the PP interval is more useful as a diagnostic indication for diabetes than the RR interval is.

### 5.1. Clinical Implications

The analysis PP intervals of the cDM model showed the alterations of ANS preventing side effects and would allow diagnosis in several stages of DM patients. The data analyzed with this method infer the development of this disease with SD1, SD2, and SD1/SD2 of heart rate variability. Additionally, with analysis of PP intervals in PSD, this showed possibility of diagnosis and early prognosis of CAN. For this reason, we propose the use of HRV for diagnosis of DM chronic in several stages; additionally, it is a noninvasive and cheap tool and has easy arithmetic calculus.

## Figures and Tables

**Table 1:** Comparison of heart rhythm between age and development of DM.

Mouse age (weeks)	Human age (years)	DM development (human time)	BPM control	BPM (cDM)
8	17-22	—	284 ± 46	
18	30-35	8-10 years	371 ± 51^∞^	313 ± 78^∞^^∗^
28	40-45	15 years	383 ± 64^∞^	405 ± 61^∞^^∗^

DM was induced in 8-week-old mice; ^∗^*μ* ± SEM vs. control; ^∞^*μ* ± SEM vs. adulthood.

**Table 2:** Alterations of HRV of frequency pacemaker by diabetes.

Frequency (ii/min)	Intrinsic activity (ms)	SD1	SD2	Poincaré index		
				SD1/SD2	Frequency	Variation CT vs. cDM
8 years' development						
CT = 218 ± 55	Interval_CT_ = 275 ± 73	10	61	0.2		
cDM = 258 ± 50^∗^	Interval_cDM_ = 233 ± 50^∗^	61^∗^	45^∗^	1.35	Increase 18%	Increase 377%
15 years' development						
CT = 190 ± 59	Interval_CT_ = 351 ± 30^∞^	15^∞^	15^∞^	1		
cDM = 208 ± 63^∗^	Interval_cDM_ = 327 ± 23^∗^^∞^	13^∗^^∞^	14^∗^^∞^	0.9	Increase 9%	—

Student *t*-test: *p* < 0.05^∗^ vs. control; Student *t*-test: *p* < 0.05^∞^ adulthood. ii: interval interpotential.

**Table 3:** Relationship HRV with age and development DM as human.

ECG (ms) interval	SD1	SD2	Poincaré index		
			SD1/SD2 ratio	Variation adulthood	Variation vs. control
Adulthood (17-22 old years)					
RR_CT_ = 218 ± 33	12	28	0.43	**—**	
PP_CT_ = 215 ± 45	19	35	0.54	**—**	
8 years' development					
RR_CT_ = 166 ± 28^**∞**^	2	28	0.07	Decrease 600%	
RR_cDM−model_ = 206 ± 62^∗^ (24%)	8^∗^	46^∗^	0.17	Decrease 260%	Increase 256%
PP_CT_ = 182 ± 45	8.2^∞^	24^∞^	0.34^∞^	Decrease 68%	
PP_cDM−model_ = 203^∗^ (12%)	10^∞^	56^∞^^∗^	0.18^∗^^∞^		Increase 188%
15 years' development					
RR_CT_ = 161 ± 30^∞^	1^∞^	1.3^∞^	0.8	Increase 86%	
RR_cDM−model_ = 151 ± 23^∗^^**∞**^ (24%)	0.6^∗^^∞^	0.6^∗^^∞^	1		Increase 25%
PP_CT_ = 158 ± 28	1.1^∞^	1.1^∞^	1^∞∞^	Increase 232%	
PP_cDM−model_ = 159 ± 29 (12%)	1.2^∞^	0.9^∞^	1.3^∗^		Increase 30-%s

Student *t*-test: *p* < 0.05^∗^ vs. control, Student *t*-test: *p* < 0.05^∞^ adulthood.

## Data Availability

The time series of ECG (PP, RR) and the nodal electrical activity (interpotential) data used to support the findings of this study are available from the corresponding author upon request to the email of Julián Torres-Jácome, PhD: jtorresjacome@gmail.com.
